# Insights and Aspects to the Modeling of the Molten Core Method for Optical Fiber Fabrication

**DOI:** 10.3390/ma12182898

**Published:** 2019-09-07

**Authors:** Maxime Cavillon, Peter Dragic, Benoit Faugas, Thomas W. Hawkins, John Ballato

**Affiliations:** 1 Institut de Chimie Moléculaire et des Matériaux d’Orsay (ICMMO), Université Paris-Sud, Université Paris-Saclay, CNRS, 91400 Orsay, France; 2 Center for Optical Materials Science and Engineering Technologies (COMSET) and the Department of Materials Science and Engineering, Clemson University, Clemson, SC 29634, USA; 3 Department of Electrical and Computer Engineering, University of Illinois at Urbana-Champaign, Urbana, IL 61801, USA; 4 Coherent Nufern Incorporated, East Granby, CT 06026, USA

**Keywords:** molten core method, dissolution, diffusion, optical fiber

## Abstract

The molten core method (MCM) is a versatile technique to fabricate a wide variety of optical fiber core compositions ranging from novel glasses to crystalline semiconductors. One common feature of the MCM is an interaction between the molten core and softened glass cladding during the draw process, which often leads to compositional modification between the original preform and the drawn fiber. This causes the final fiber core diameter, core composition, and associated refractive index profile to vary over time and longitudinally along the fiber. Though not always detrimental to performance, these variations must, nonetheless, be anticipated and controlled as they directly impact fiber properties (e.g., numerical aperture, effective area). As an exemplar to better understand the underlying mechanisms, a silica-cladding, YAG-derived yttrium aluminosilicate glass optical fiber was fabricated and its properties (core diameter, silica concentration profile) were monitored as a function of draw time/length. It was found that diffusion-controlled dissolution of silica into the molten core agreed well with the observations. Following this, a set of first order kinetics equations and diffusion equation using Fick’s second law was employed as an initial effort to model the evolution of fiber core diameter and compositional profile with time. From these trends, further insights into other compositional systems and control schemes are provided.

## 1. Introduction

The molten core method (MCM) has now been employed for ~25 years to produce “nonconventional” optical fibers with intriguing properties [1], including crystalline semiconductors (Si, Ge) core–silica (SiO_2_) cladding [2], and low optical nonlinearity multicomponent fluorosilicate core–silica cladding [3] glass, to name just a few. Most optical fibers fabricated using the MCM are silica-clad, that is, the preform is a fused silica capillary tube and will serve as the fiber cladding material post draw.

One intrinsic feature of such fibers fabricated using the MCM is the inherent incorporation of SiO_2_ into the (molten) core as the fiber is being drawn. This SiO_2_ incorporation typically is accompanied with a proportionate decrease of the fiber core diameter and the establishment of a fiber core graded index profile [4,5]. In most cases, some mixing between SiO_2_ from the preform and the molten precursor is desired as it facilitates the formation of a glassy core during fiber drawing. However, the kinetics of SiO_2_ incorporation are so rapid (typically > 60 mol % within 10–20 min) that it usually is seen as inevitable. A direct consequence to this dynamic SiO_2_ incorporation during the draw is the difficulty to design fibers with well-controlled properties (e.g., mode area, numerical aperture). Additionally, very high (>40 mol %) dopant (i.e., non-silica) concentration in the fiber core is challenging to achieve, somewhat limiting fiber development such as Zero Brillouin Activity (ZeBrA) glass optical fibers [6].

With this in mind, this work provides some insights into the mechanisms that drive this SiO_2_ incorporation. In this context, a yttrium aluminosilicate core–silica glass optical fiber is fabricated using the MCM, and segments are collected at different positions along the drawn fiber length and characterized using scanning electron microscopy and energy dispersive x-ray analysis. By comparing the segments, along with data available from the literature, it is concluded that SiO_2_ incorporation results from a diffusion-controlled dissolution process. Following this discussion, a set of first order kinetics and diffusion equations are introduced to model the observed “typical features” of the MCM. It should be specified that this work is a first attempt at describing the MCM process through a modeling aspect, and, therefore, multiple assumptions are made. However, it is anticipated that future developments based on this model will consider more complex aspects of the fiber draw process not taken into account herein, such as the specific temperature distribution of the draw furnace, the heating element used, the neckdown preform shape (and temperature profile associated with it), and/or the presence of convective flow induced during drawing.

## 2. Materials and Methods

The molten core method can generally be described as follows. First, a precursor material (e.g., a crystal rod, a powder mixture, or ceramic pellets) is introduced inside a glass capillary tube preform having one end closed. This preform then is placed inside a furnace and is heated to high temperatures, typically ~2000 °C for a fused silica capillary tube. At this temperature, the precursor melts and the glass (silica for the purposes of this work) cladding softens, enabling the preform to be drawn into a glass clad optical fiber. During the time that the molten core is in the furnace, it is in direct contact with the inner walls of the (silica) cladding tube and, subsequently, species from the cladding are incorporated into the melt. Understanding the nature of this interaction/incorporation is one of the motivations of this work. 

In this work, a 2 weight percent (wt %) erbium-doped yttrium aluminum garnet (Er:YAG, Northrop Grumman Synoptics, Charlotte, NC, USA) single crystal rod of 1.5 mm diameter and 20 mm length was chosen as an initial precursor material. It was drawn at 2050 °C inside a fused silica capillary preform (3 mm inner and 30 mm outer diameters), with a targeted fiber diameter of 125 μm drawn at both constant feed (0.5 mm/min) and draw (30 m/min) speed. This Er:YAG crystal precursor was selected because: (i) It is a fully densified rod with well controlled geometry, ii) it is a well-known material, and the authors have had extensive experience in drawing such YAG-derived fibers [7,8], and (iii) YAG has a melting temperature of ~1940 °C, well below the fiber drawing temperature and therefore the precursor is fully melted at the draw temperature. It is worth mentioning that the YAG crystal was doped with 2 wt % of erbium, but this relatively small amount is assumed to have a neglectable influence on the melting and drawing conditions with respect to a pure (undoped) YAG rod. 

A total of ~1 km of fiber was drawn, and four fiber segments were collected at different positions along the fiber, equating to different amounts of time that the molten core/softened glass cladding had to interact, and analyzed using energy dispersive x-ray (EDX) spectroscopy coupled with scanning electron microscope (SEM; HITACHI-6600, Hitachi High Technologies in America, Schaumburg, IL, USA) using an accelerating voltage of 20 kV. This analysis enables the knowledge of both concentration profiles and fiber core diameters. The first segment was collected after ~10 min of drawing, which corresponds to the shortest amount of time needed for the system to stabilize and to spool the fiber with a fairly good control of the fiber cladding diameter. The core and cladding diameters were measured from the SEM micrographs. The modeling work is described and discussed in the next section.

## 3. Results

### 3.1. Fiber Drawing and General Insights

**Figure 1:**
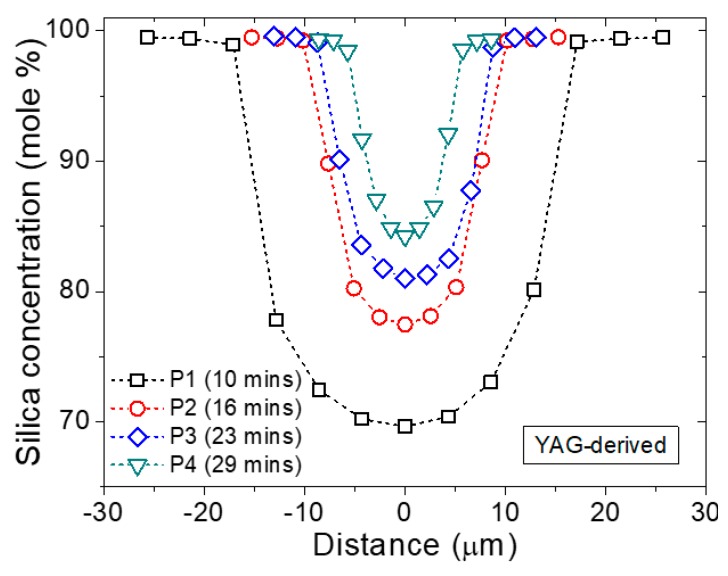
Electron Dispersive X-ray (EDX) elemental analysis of the silica concentration (in mol %) as a function of core radial position for a YAG-derived optical fiber fabricated using the Molten Core Method (MCM), and for various fiber segments collected at different times during fiber drawing.

In Figure 1, the SiO_2_ radial composition profiles for the 4 segments investigated are displayed. Additionally, several typical fiber parameters are reported in Table 1. At this point, it is worth noting that this work focuses on SiO_2_ incorporation, and therefore the composition profiles of the other elements are left out of the present analysis. The principal time-related characteristics of the MCM fiber fabrication process clearly identified from Figure 1 are:

(i) The first fiber segment of the draw already exhibits a graded-index profile with a lower concentration of SiO_2_ at its core center relative to later segments. This concentration increases over time. As opposed to this, the initial precursor composition decreases (not presented here) as it is diluted by the SiO_2_.
(ii) For a constant fiber cladding diameter, the fiber core diameter decreases as the fiber is being drawn and, therefore, the initial inner diameter / outer diameter (ID/OD) ratio is not preserved during the draw. Moreover, it is worth noting that the initial segments collected generally present a larger ID/OD ratio with respect to the initial ratio (e.g., 2/10 versus 1/10 here).
(iii) The concentration profile sharpens over time, i.e., the silica composition profile is less flat at the core center as draw time increases. This concentration profile is indicative of a diffusion-controlled incorporation of silica into the molten core.

In view of the sharp core/cladding interface observed in Figure 1 (but also well visible from refractive index profiles in these systems [7]), coupled with the reduction of the core diameter, it appears that the silica species principally diffuse into the molten core precursor, and not the other way around (i.e., core species diffusing into the SiO_2_ cladding). In order to gain better insights into why silica is incorporated into the molten core, one may consider the dissolution of a solid rod into a “low” viscosity melt, as it has extensively been investigated in the literature (e.g., [9,10,11,12]). In these studies, a rod material (the solute) was progressively dissolved into a melt/slag (the solution) and, then, diffused into it through a diffusion-controlled dissolution mechanism. However, it must be pointed out that in each studied case, the precursor (e.g., sapphire [9], MgO [12]) remained well below its melting temperature. In the present case, at the temperature achieved during the molten core method (typically ~2000 °C), the situation is different. Both the precursor and the silica glass are in a molten state, and the precursor is brought well above its melting temperature (by ~100 K in the present study). Because of this particularity, here the precursor becomes the low viscosity solution into which the higher viscosity fused silica (the solute) dissolves. To put this into perspective, Table 2 reports selected viscosity values for materials of interest at various temperatures, and additional viscosity data can be found in [13] for a large number of aluminosilicate systems. 

From Table 2, it can be seen that the molten core precursors (and many silicate systems, again from Ref. [13]) exhibit low viscosities of, at most, a few hundreds of Pa·s, and generally are several order of magnitudes lower than the viscosity of molten silica at the draw temperature. It is interesting to note that, as the temperature increases, the difference in viscosity values in aluminosilicate systems is greatly reduced [13]. On a lighter, but no less informative note, the viscosity of molten Al_2_O_3_ or YAG at the fiber draw temperature is only × 10 higher than cold (12 °C) wine [17]. This surely depicts the “liquid” character of the molten core compared to its surrounding silica cladding. However, it is evident that the progressive incorporation of SiO_2_ into the molten core (>60 mol % in 10 min under the draw conditions employed here) would increase the viscosity. 

In this section typical characteristics of the MCM draw were discussed. From this discussion the low viscosity of the precursor in the molten state with respect to the silica cladding was highlighted. Following this, during the fiber drawing process, the molten core precursor can be considered the solution and the silica from the surrounding preform the solute. This intermediate conclusion sets the basis for the modeling section that follows.

### 3.2. Preliminary Assumptions and Modeling Equations

The fiber draw process is first addressed and discussed in view of making several assumptions and simplifications. The draw process is separated into three steps (above, inside, and below the heat-zone, respectively):

*Above the heat-zone*: The preform, comprising the precursor core inside the glass cladding, is placed inside the draw furnace and, while inside, remains above the heating element or heat-zone (i.e., the precursor is in the solid state and has not yet melted). The fiber draw temperature is assumed to be reached only inside the heat-zone, and, therefore, above the heat zone, the temperature is below the actual furnace setpoint such that dissolution is assumed here to be negligible. In [18], diffusion at a sapphire/fused SiO_2_ interface for 4 h at 1800 °C (i.e., below the sapphire melting point) yielded an aluminosilicate layer of only ~300 μm thickness. Here, the timescale is much shorter (~10 min, i.e., × 24 shorter) and the solid rod interface is not always in contact with the silica preform as it sits inside the silica capillary. Consequently, it is reasonable to neglect any interaction occurring between the silica glass and the precursor at this stage. However, and for completeness, it is worth pointing out that in the case of powder mixtures and low melting temperature precursors (e.g., fluorides such as SrF_2_ as in Ref. [3]), one may expect interactions/reactions to take place in the furnace as the sample approaches the heat zone. This is not the case in this present study. Additionally, formation of intermediate low melting temperature phases can further complicate the analysis and are ignored herein.

*Inside the heat-zone*: When the desired draw temperature is reached (in the present case, 2050 °C), the preform is lowered into the heat-zone (40 mm long here). Since, in this region, the temperature is now above the melting temperature of the precursor, the latter is expected to melt shortly thereafter. For a silica preform of 30 mm outer diameter, the preform diameter entering the heat zone is ~11 mm, and the preform diameter exiting the heat zone (and hence the furnace) is 125 μm, i.e., the size of the fiber. The previous value of ~11 mm was determined experimentally from observation of the preform neckdown post-draw and is shorter than the initial 30 mm preform OD. This likely is due to the silica tube narrowing inside the furnace, although still above the heat zone element. It is worth pointing out that the YAG crystal has an OD of 1.5 mm, which is smaller than the initial 3 mm preform ID, permitting the tapering to take place while the precursor crystal remains in its solid state. At the heat-zone entrance, the silica tube ID should be 1.5 mm (the diameter of the YAG rod) while its OD is 11 mm, and therefore the ID/OD is found to be ~0.14 (1.5 mm/11 mm), close but slightly higher than the original value of 0.1 corresponding to the 3 mm/30 mm dimension of the silica tube preform. Further investigations are necessary to better comprehend how the precursor material influences the ID/OD preform ratio as it enters the heat-zone and progressively melts. It is in this “active region” (i.e., the heat-zone) that silica incorporation into the molten core mostly takes place. At this point, the concept of an effective diameter, *d_eff_*, is introduced, and will be used later to set the modeling equations. The effective diameter is defined as the averaged inner preform diameter within the heat-zone (again which corresponds to the active region). In this experiment, the preform outer radius at the heat-zone center is ~5.5 mm (i.e., 11/2 mm assuming a cone-like shape). By taking the preform ID/OD = 0.14 ratio to be constant in this region, then *d_eff_* = 700 μm (at *t* = 0), and the effective radius *R_eff_* = 350 μm (also at *t* = 0) will be later used in the modeling section. Introducing *d_eff_* (and *R_eff_*) has the advantage of simplifying the modeling as will be seen later. Additionally, a constant temperature is assumed throughout the heating element, which is also set for simplicity.

*Below the heat-zone*: The fiber has reached its final targeted geometry (e.g., 125 μm cladding diameter), and quickly exits the furnace (speed of ~30 m/min) where the glass is quenched and, consequently, any further dissolution of SiO_2_ is ceased.

Additional assumptions are made when considering interaction between SiO_2_ and the precursor inside the heat-zone. First, the diffusion coefficient (*D*) of SiO_2_ into the molten core is assumed constant throughout the draw. This certainly introduces further uncertainties since *D* is a function of viscosity/composition, and the composition of the molten core changes as a function of time. However, since, at these high temperatures the viscosity values somewhat converge as discussed above, the impact of compositional change on *D* is assumed to be small. Second, free and forced convective flows are ignored and left for future modeling work as it brings additional complexity.

Now let us consider that the radial compositional profile for each fiber core segment collected is a function of time (*t*), core radius (*R*), and silica concentration at the core center (*c_m_*). Additionally, as was discussed previously, the core diameter reduction was associated with an increase in silica concentration, and, from this observation, it becomes evident that these two features are related to each other. Therefore, *dR/dt* and *dc_m_/dt* (variations of *R* and *c_m_* with respect to time, respectively) must follow a similar dependency. Here, first order kinetics equations are employed, which originate from the well-known Noyes-Whitney equation [19], and are employed herein to describe the dissolution kinetics of cladding silica into the molten core. Because it was stated above that the process is diffusion-controlled, the Noyes-Whitney equation can be transformed into the Nernst–Brunner equation [20], and this is the final form that will be used (Equations (1) and (2)). Additionally, the silica concentration profile in the fiber core can be considered using Fick’s second law in cylindrical coordinates (Equation (3)) [21]. Thus, one gets the following equations:

(1) $$\frac{dR}{dt}=k\left(R_{s}-R\right),\;and\;k=\frac{SD}{VR_{eff}}=\frac{\left(2\pi R_{eff}L\right)D}{\left(\pi R_{eff}^{2}L\right)R_{eff}}=\frac{2D}{R_{eff}^{2}}$$

(2) $$\frac{dc_{m}}{dt}=k\left(c_{s}-c_{m}\right)$$

(3) $$\frac{\partial c_{R}}{\partial t}=\frac{D}{r}\frac{\partial }{\partial r}\left(r\frac{\partial c_{R}}{\partial r}\right)$$

By integrating Equations (1)–(3), one obtains:

(4) $$R=(R_{0}-R_{s})e^{-kt}+R_{s}$$

(5) $$c_{m}=c_{s}\left[1-e^{-kt}\right]$$

(6) $$c_{R}\left(t,\;r\right)=\left[1-2\overset{\infty }{\underset{n=1}{\sum }}\frac{e^{\frac{-D\alpha_{n}^{2}t_{x}}{R_{eff}^{2}}J_{0}\left(\frac{\alpha_{n}r}{R_{eff}}\right)}}{\alpha_{n}J_{1}\left(\frac{\alpha_{n}r}{R_{eff}}\right)}\right]\left(c_{surf}-c_{m}\right)+c_{m}$$

To help the reader, all the parameters used in Equations (1)–(6) are summarized in Table 3.

The silica composition profile (*c_R_*) is calculated from *r* = 0 to *r* = *R_eff_*, and then scaled to the final fiber core geometry. However, *R_eff_* (Equations (4) and (5)) is also a function of time and was found to slightly decrease (~15% within ~2000 s) after analyzing the preform post draw. This decrease of *R_eff_* is assumed to depend linearly with time and, thus, *R_eff_* (*t*) = *R_eff_* (*t* = 0) − 2.625 × 10^−6^ × *t*. Further analysis should permit a better understanding of the time dependence of the effective preform radius. Additionally, *t_x_* (in Equation (6)) corresponds to the time spent by the preform inside the heat-zone. This time is very sensitive to the preform shape, draw furnace hot-zone thermal profile, feed and draw speeds, and can range from tens of seconds to several minutes. Here, assuming a constant feed rate of 0.5 mm/min, *t_x_* is estimated to be ~40 s.

### 3.3. Modeling Results and Discussion

**Figure 2:**
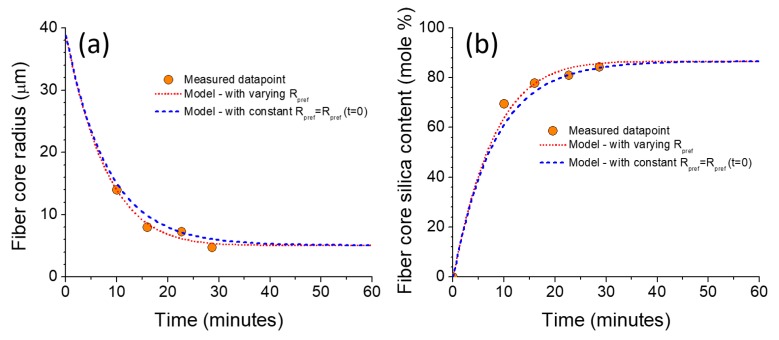
(**a**) Evolution of fiber core radius and (**b**) fiber core silica content as a function of draw time. Fit (red curve in (**a**)) performed using Equation (4) to determine *R_0_, R_s_*, and *D*. These parameters are thus used to fit the red curve in (**b**). More details can be found in the text.

Equation (4) first was used to fit the core radius data of the four fiber segments and the results are reported in Figure 2a. Here *R_eff_* (*t* = 0) is set to 0.0350 cm (i.e., *d_eff_*/2), which, once again, corresponds to the averaged effective radius, and *R_0_*, *R_s_*, and *D*, are fit parameters. The fit is performed, and results are displayed in Figure 2a (red curve). From the fit, one obtains a diffusion coefficient of SiO_2_ into the melt of D = 1.25 × 10^−6^ cm^2^/s, along with *R_0_* = 38.9 μm, and *R_s_* = 5.1 μm. By keeping these values constant but taking *R_eff_* at any time t to be the constant value *R_eff_* (*t* = 0), the blue curve in Figure 2a is obtained. There is no significant difference between the two cases, and one may simply use a constant *R_eff_* as a first approximation. Now, if the core compositions at the fiber segment core centers are fitted using Equation (5), one finds D = 1.67 × 10^−6^ cm^2^/s, and *c_s_* = 82.9 mol %. It is interesting to point out that this value of D is found to be somewhat similar to the previously calculated value (D = 1.25 × 10^−6^ cm^2^/s), and tends to indicate that the two processes, namely silica diffusion and diameter reduction, are directly related to each other, as suggested above and not entirely unexpected. The diffusion coefficient values found here fall within the same order of magnitude as what has been reported in the literature (e.g., Refs. [22,23]). Consequently, D = 1.25 × 10^−6^ cm^2^/s is used throughout the rest of this analysis. Employing this later D value in Equation (5) to fit the data (Figure 2b), one obtains *c_s_* = 86.6 mol %, which is reasonably close to the previously obtained value of 82.9 mol %. Again, in Figure 2b one can note that by holding *R_eff_* constant throughout the modeling, only a slight deviation is noticed with respect to a varying *R_eff_*.

**Figure 3:**
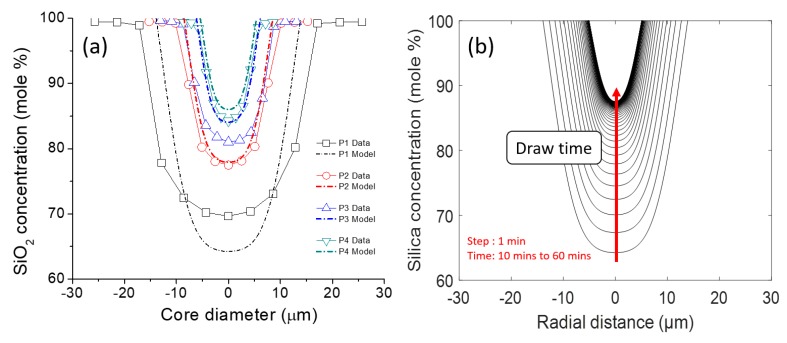
(**a**) Comparison between modeled data and measured data (using Equations (4)–(6) from the text). (**b**) Evolution of silica concentration profile as a function of time with a time step interval of 1 min.

Now that *D*, *c_m_ (t),* and *t_x_*, are calculated, the silica composition profile for each fiber segment can then be computed using Equation (6). Thus, combining Equations (4)–(6) together, the complete silica composition profile at any given time can be estimated. In Figure 3a, the computed profiles are compared to the measured ones from EDX, and, in Figure 3b, the profiles are computed for a 50 min time lapse (10 min to 60 min of draw with a 1-minute time interval). In Figure 3a, a core diameter mismatch between calculated and measured profiles can be observed. Additionally, the measured profiles typically exhibit higher SiO_2_ concentration with respect to the computed ones. This is attributed to the electron beam probe radius being on the order of few μm, thereby shifting the concentration profiles to higher SiO_2_ contents and larger apparent core radii. Besides these mismatches, the general MCM draw features as discussed above are well visible.

These results demonstrate that by simply knowing (or assuming) *c_s_* and with knowledge of core diameters over a fairly large range of drawn fiber length, silica concentration profiles during the entire draw range can be computed. This work, therefore, is expected to ease the future development of optical fibers when the MCM is employed. 

**Figure 4:**
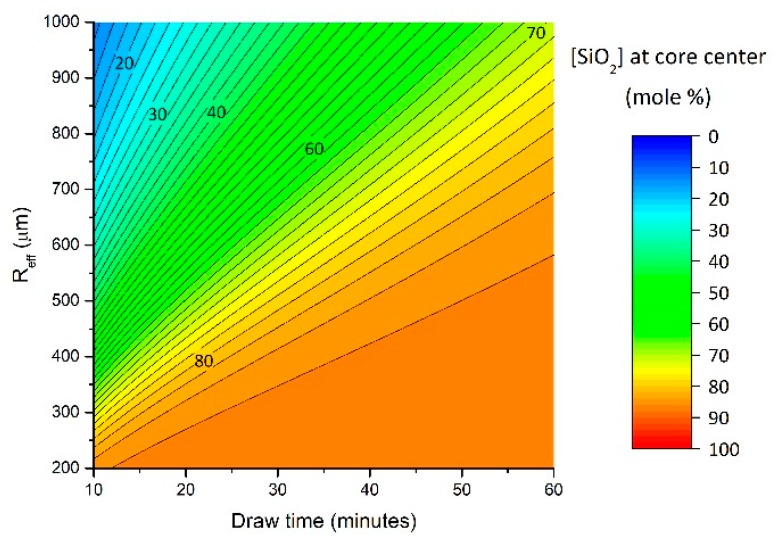
Changes in SiO_2_ concentration at the final fiber core center as a function of the effective radius (*R_eff_*) during fiber drawing. Fiber is collected typically 10–15 min after the sample is in the furnace, which sets the lowest draw time bound.

Additionally, the model presented here can make interesting predictions, although these need to be further validated. For instance, *R_eff_* factors into Equations (4)–(6). One may wonder how the silica concentration at the fiber core center would evolve if the initial *R_eff_* value was modified. To answer this question, the model was used and *R_eff_* is varied (from 0.2 mm to 1 mm), everything else being held constant. The results are reported in Figure 4, and clearly suggests maximizing *R_eff_* would yield reduced silica concentration into the final fiber core. As an example, after ~20 min of draw time, the SiO_2_ concentration at the fiber core center is expected to change from ~80 mol % to ~70 mol % when *R_eff_* varies from 350 μm to 450 μm. Here, the authors would like to emphasize that further experiments must be carried out to further validate these expected trends, as other factors not yet accounted for and presently ignored may play a dominant role.

Based on the above results, it is suggested that drawing a cane instead of an optical fiber would also increase *R_eff_*, thus increase the volume in which silica has to diffuse, and, consequently, decrease the silica concentration in the resultant molten core at a given length. On the other hand, increasing the fiber outer cladding size with constant feed speed would result in a longer time spent by the precursor in the furnace. These two effects are expected to somewhat counteract each other. Increasing the feed speed (hence the draw speed) would promote “step-index like” fiber profiles, as *t_x_* (the time spent by the molten precursor in the heat-zone) would be reduced. To further control silica incorporation, a post-feeding molten core method can be employed as in [5], in which a precursor is added during fiber drawing. Finally, the effect of temperature has not been discussed. It is fair to ask to what extent changing the drawing temperature (e.g., 2000 °C versus 2100 °C) would impact the silica dissolution rate. Obviously the silica cladding would exhibit a different viscosity (Table 2) and this would somehow impact the rate at which silica diffuses into the fiber core. An interesting future step would be to investigate and clarify such temperature effects.

## 4. Conclusions

This paper provides a first investigation into the evolution of properties—specifically core diameter, silica concentration and its associated graded index profile—of fibers drawn using the molten core method (MCM). In this study, an yttrium aluminosilicate core–silica cladding glass optical fiber was fabricated, and its properties investigated at different positions along the drawn fiber length. A diffusion-controlled dissolution mechanism is suggested as the driver for incorporation of silica into the fiber core during the fiber fabrication process and was modeled using first order kinetics and diffusion equations. Provided the assumptions made in this preliminary work, this simulation was in rather good agreement with experimental data. Fibers are currently developed using larger inner diameter capillary preforms, and along with collection of more data in various drawn systems (including fluorosilicates), the proposed model is expected to be further strengthened. In this work, only the evolution of silica concentration has been investigated, and future modeling work will include the evolution of dopant concentrations (i.e., Y and Al).

## Figures and Tables

**Table 1:** Fiber segments collected during the draw and their respective properties (composition is in mol %).

Segment	Position during Draw (m)	Time during Draw (min)	Core/Cladding Diameters	SiO_2_	Al_2_O_3_	Y_2_O_3_	Er_2_O_3_
P1	0	10.0	28/136	69.6	19.1	11.1	0.2
P2	180	16.0	16/123	77.5	13.2	9.1	0.2
P3	380	22.7	14.5/128	81.0	10.9	7.9	0.2
P4	560	28.7	9.5/128	84.3	9.1	6.5	0.1

**Table 2:** Viscosity values for various molten materials.

Material	Viscosity (Pa.s)	References
SiO_2_ (2000 °C)	~45,000 ^a^	[14]
SiO_2_ (2100 °C)	~15,000 ^a^	[14]
1Al_2_O_3_-3SiO_2_ (2000 °C)	~15,000 ^b^	[14]
1Al_2_O_3_-1SiO_2_ (2000 °C)	~0.5 ^b^	[14]
3Al_2_O_3_-1SiO_2_ (2000 °C)	~0.1 ^b^	[14]
Al_2_O_3_ (2120–2220 °C)	~0.03–0.025	[15]
YAG (1970–2070 °C)	~0.04–0.045	[16]
Pinot Blanc (12 °C)	~0.003	[17]

^a^: Viscosity values calculated from Equation (5) in Ref. [14]. ^b^: Viscosity values deduced from Figure 5 in Ref. [14].

**Table 3:** Parameters used during modeling and their associated meaning/definition.

Parameter Symbol	Description/Definition
*R*	Fiber core radius at time *t*.
*R*_0_	Initial fiber core radius (at time *t* = 0 s)
*R_s_*	Fiber core radius at saturation, i.e., over long length
*R_eff_*	Effective preform radius in the active region, *R_eff_* = *d_eff_*/2
*k*	SiO_2_ dissolution constant, $k=\frac{SD}{VR}$, in·s^−1^
*S*	Surface of the active region, $S=2\pi R_{eff}L$
*D*	Diffusion coefficient, in cm^2^·s^−1^
*V*	Volume of the active region, $V=\pi R_{eff}^{2}L$
*L*	Heat-zone length (40 mm)
*c_m_*	Silica concentration (in mol %) at the fiber core center
*c_s_*	Silica concentration (in mol %) at saturation, i.e., over long length
*r*	Radial distance from center (*r* = 0) to the effective preform radius (*r* = *R_eff_*)
*c_R_*	Silica concentration (in mol %) along the radial profile
*c_surf_*	Silica concentration (in mol %) at the core/cladding interface, and set to 100%
*t_x_*	Time for the preform to go through the active region
*J*_0_, *J*_1_, *α_n_*	Bessel functions of the first kind of zeroth order (*J*_0_) and first order (*J*_1_); *α_n_* is the *n*-th zero of *J*_0_
